# CRISPR-Mediated *POSTN* Editing Modulates Proliferation, Apoptosis, and Molecular Profiles of Primary Rabbit Hair Follicle Stem Cells via the cAMP/PKA/CREB Signaling Pathway

**DOI:** 10.3390/cells15171516

**Published:** 2026-08-23

**Authors:** Jiawei Cai, Bohao Zhao, Aoyun Fan, Yang Chen, Xinsheng Wu

**Affiliations:** 1 College of Animal Science and Technology, Yangzhou University, Yangzhou 225009, China; cjwcjw520800@163.com (J.C.); bhzhao@yzu.edu.cn (B.Z.); 17637894476@163.com (A.F.); yangc@yzu.edu.cn (Y.C.); 2 Joint International Research Laboratory of Agriculture & Agri-Product Safety, Yangzhou University, Yangzhou 225009, China

**Keywords:** POSTN, CRISPR/Cas9, hair follicle stem cells, transcriptome, metabolomics

## Abstract

Hair follicle stem cells (HFSCs) are critical for hair follicle (HF) morphogenesis and cyclic hair regeneration. Periostin (POSTN), an extracellular matrix protein involved in tissue development and skin cell regulation, remains poorly characterized in HFSCs. In this study, we constructed CRISPR/Cas9 vectors targeting *POSTN* and validated their editing efficiency in primary HFSCs. *POSTN* editing significantly suppressed cell proliferation and promoted apoptosis. Transcriptomic analysis identified 988 differentially expressed genes (DEGs) enriched in immune responses and MAPK, PI3K–Akt, and cAMP pathways. Metabolomic analysis revealed 98 differential metabolites (DMs) associated with nucleotide metabolism and FoxO, AMPK, and cAMP pathways. Integrated multi-omics analysis showed extensive correlations between DEGs and DMs, and highlighted the cAMP pathway as the core regulatory axis. Western blot (WB) validation confirmed that *POSTN* editing reduced PKA and CREB phosphorylation, indicating inhibition of the cAMP/PKA/CREB signaling. These findings demonstrate that POSTN regulates HFSCs’ proliferation and apoptosis partially via the cAMP/PKA/CREB pathway, providing novel insights into the functional regulation of HFSCs.

## 1. Introduction

Hair follicles (HFs) are important accessory organs of mammalian skin that undergo cyclic regeneration throughout life. This process is driven by the periodic and orderly activation of hair follicle stem cells (HFSCs), which enables HFs to progress through the anagen, catagen, and telogen phases of the hair cycle [1]. HFSCs are located in the bulge region of the HF and possess strong self-renewal capacity and multilineage differentiation potential, making them the core cell population responsible for maintaining hair follicle regeneration and skin homeostasis [2,3,4]. The biological behavior of HFSCs is tightly regulated by their specialized niche, which consists of multiple cell types and abundant extracellular matrix (ECM) components that together determine their fate [1,5]. Therefore, elucidating the molecular mechanisms that regulate HFSC homeostasis is essential for understanding HF development and cyclic regeneration.

Periostin (POSTN) is a secreted ECM protein predominantly expressed in collagen-rich connective tissues. It is widely involved in numerous biological processes, including tissue development, wound healing, cell proliferation, differentiation, and extracellular matrix remodeling [6]. POSTN interacts with multiple ECM components, including collagen and fibronectin, contributing to the maintenance of extracellular matrix structure [7]. During tissue injury, POSTN expression is significantly upregulated. Studies have shown that the skin wound closure rate and healing efficiency in *POSTN* knockout mice are significantly lower than those in wild-type (WT) controls [8,9]. POSTN specifically binds to integrin receptors on the cell membrane surface, including αvβ3 and αvβ5, to activate intracellular signaling pathways that regulate cell proliferation, migration, and differentiation [7]. Furthermore, *POSTN* upregulates IL–6 expression through fibroblast-derived paracrine signaling, indirectly stimulating keratinocyte proliferation [10]. Activation of the NF–κB signaling pathway by POSTN has also been reported to facilitate keratinocyte proliferation and HF regeneration following skin injury [9]. In recent years, some studies have shown that *POSTN* is a key differentially expressed gene in scalp tissue of patients with androgenetic alopecia (AGA), suggesting that it may be involved in the pathological process of hair-related diseases [11]. Although the biological function of POSTN in skin repair has been well elucidated, its regulatory effect on the function of HFSCs and its potential molecular mechanism remain to be elucidated.

This study employed primary HFSCs to establish a *POSTN*-edited cell model using CRISPR/Cas9 genome-editing technology. Functional analyses, integrated transcriptomic profiling, and metabolomic analyses were then performed to systematically investigate the molecular mechanisms by which POSTN regulates HFSCs’ biological functions. This study provides new insights into the role of POSTN in HF biology and identifies potential molecular targets for future investigations of HF regeneration.

## 2. Materials and Methods

### 2.1. Animals

Twelve 6-month-old male Angora rabbits were selected for the isolation of primary HFSCs from dorsal skin. All rabbits were housed in the same rabbit facility with uniform environmental conditions, and cared for by a dedicated animal technician in accordance with routine husbandry practices of commercial rabbit farms. Each rabbit was housed individually in a single cage, fed manually twice daily (morning and afternoon), and provided with ad libitum access to water. Formal experiments were conducted after a 1-week acclimatization period. Before tissue collection, rabbits were anesthetized by intravenous administration of Zoteil–50 (5 mg/kg) via the marginal ear vein. A 1 cm^2^ full-thickness dorsal skin sample was excised under aseptic conditions, and the wound was immediately disinfected with iodophor. After that, the mental state, feeding and drinking water, and wound healing of each animal were monitored daily for 7 consecutive days, and no serious symptoms were observed. All experimental procedures were approved by the Animal Ethics Committee of Yangzhou University (Approval No. 202205123).

### 2.2. Cell Isolation, Culture, and Transfection

Primary HFSCs were isolated from rabbit dorsal skin, and the specific procedures are as follows: the skin samples were immersed in 75% ethanol for 1–2 min, and then washed three times with phosphate-buffered saline (PBS) (BL302A, Biosharp, Beijing, China) containing 1% penicillin–streptomycin (15140122, Gibco, Grand Island, NY, USA). After the subcutaneous adipose tissue was carefully removed, it was digested in 0.1% neutral protease II (Dispase II) (04942078001, Sigma, St. Louis, MI, USA) for 2 h. After digestion, a single hair follicle was isolated by microdissection. The isolated hair follicles were digested with 0.25% Trypsin–EDTA (25200072, Gibco, Grand Island, NY, USA) at 37 °C for 30 min, and then the same volume of complete medium was added to terminate the digestion. The cell suspension was filtered through a 70 μm filter membrane and centrifuged at 1000 rpm for 10 min. The cells were resuspended in the basic medium of hair follicle stem cells (S021-002B, iCell, Shanghai, China). After precipitation, the cells were inoculated into a Petri dish and cultured in an incubator at 37 °C and 5% CO_2_ [12]. After 24 h of culture, the fresh medium was replaced to remove the unattached miscellaneous cells and complete the preliminary purification of the cells. Passage 2 (P2) cells were used for subsequent experiments after cell identification. For transfection, cells were seeded into six-well plates and cultured until they reached approximately 80% confluence. Plasmid transfection was performed using TransIT–X2^®^ Transfection Reagent (MIR 6000, Mirus Bio, Madison, WI, USA) according to the manufacturer’s protocol. In order to evaluate the role of the cAMP signaling pathway, *POSTN*-edited HFSCs were treated with Forskolin (HY-15371, MedChemExpress, Monmouth Junction, NJ, USA) for 24 h before collecting samples for downstream analysis.

### 2.3. Construction of the POSTN Editing Vector

Single-guide RNAs (sgRNAs) targeting exon 5 of the *POSTN* gene were designed using the CRISPOR online platform (http://crispor.tefor.net/; accessed on 8 March 2026). Three candidate sgRNAs with high targeting efficiency and low off-target potential were selected (Table 1). Complementary oligonucleotides corresponding to sgRNA were annealed and ligated into the PX458 vector to construct three recombinant editing plasmids (sgRNA–1, sgRNA–2, and sgRNA–3). Following transfection, green fluorescent protein (GFP)-positive cells were sorted using a FACSAria III flow cytometer (Becton, Dickinson and Company, New Jersey, USA). Genomic DNA was extracted using a DNA extraction kit (DP304, Tiangen Biotech, Beijing, China). Target regions were subsequently amplified by PCR, subjected to TA cloning, and analyzed by Sanger sequencing to evaluate genome-editing efficiency.

### 2.4. Thymine–Adenine (TA) Clone and Sequencing Analysis

Target genomic fragments were amplified using the primers listed in Table 2. Purified PCR products were ligated into the pCE2 TA/Blunt-Zero Vector (C601, Vazyme, Nanjing, China) and transformed, and individual colonies were selected for Sanger sequencing. Sequencing results were aligned with the WT sequence to determine the types of editing events. Gene-editing efficiency was calculated as follows:

Gene-editing efficiency (%) = (Number of mutant colonies/Total number of sequenced colonies) × 100%.

Sequencing primer sequences are provided in Table 3.

### 2.5. CCK–8 Assay

Cell viability was assessed using a Cell Counting Kit–8 (CCK–8) (A311, Vazyme, Nanjing, China). Briefly, HFSCs were seeded into 96-well plates at a density of 1 × 10^4^ cells per well 24 h after transfection. Absorbance at 450 nm was detected at 0, 24, 48, and 72 h using an Infinite M200 Pro multifunctional microplate reader (Tecan, Grödig, Austria) [13].

### 2.6. Cell Proliferation EdU Assay

HFSCs were seeded into 6-well plates at a density of 5.0 × 10^5^ cells per well. After washing with PBS, the culture medium was replaced with fresh medium containing 10 μmol/L EdU (C0075S, Beyotime, Beijing, China), and cells were incubated for an additional 2 h. After removal of the medium, cells were fixed with 4% paraformaldehyde (P0099, Beyotime, Beijing, China) at room temperature for 30 min, followed by permeabilization with 0.3% Triton X-100 (P0096, Beyotime, Beijing, China) for 15 min. After washing with PBS, 0.5 mL of Click reaction solution was added to each well, and cells were incubated in the dark for 30 min. Following washing steps, cell nuclei were counterstained with Hoechst 33342 (C1025, Beyotime, Beijing, China). Cells were observed and imaged under a fluorescence microscope (Olympus Corporation, Tokyo, Japan). Six random fields of view per sample were captured at 100× magnification. Image processing and quantification were performed using ImageJ software (Version 1.53k, NIH, Bethesda, MD, USA) following a standardized protocol. The proliferation index was calculated as the percentage of EdU-positive cells among the total cell population [14].

### 2.7. TUNEL Assay

HFSCs were seeded into 6-well plates at a density of 5.0 × 10^5^ cells per well. Cell apoptosis was detected using the One-Step TUNEL Apoptosis Assay Kit (C1089, Beyotime, Beijing, China). After fixation with 4% paraformaldehyde at room temperature for 30 min, cells were permeabilized with 0.3% Triton X-100 at room temperature for 10 min. After washing three times with PBS, TUNEL detection working solution was added, and cells were incubated at 37 °C in the dark for 1 h. After incubation, cells were washed with PBS, and DAPI working solution (C1006, Beyotime, Beijing, China) was added to counterstain cell nuclei at room temperature in the dark for 10 min. Fluorescence imaging was performed in a blinded manner by an investigator unaware of group allocation. Six random fields per sample were captured at 200× magnification. Subsequent image quantification was also conducted in a blinded fashion. TUNEL-positive cells were counted using the cell counter tool of the ImageJ software (Version 1.53k, NIH, Bethesda, MD, USA), and the apoptosis rate was calculated as follows: apoptosis rate = (number of TUNEL-positive cells/total number of cells) × 100% [15].

### 2.8. RNA Isolation and Real-Time Quantitative PCR (RT-qPCR)

Total RNA was extracted using the SteadyPure RNA Kit (AG21024, Accurate Biotechnology, Changsha, China). Complementary DNA (cDNA) was synthesized using a reverse transcription kit (AG11728, Accurate Biotechnology, Changsha, China). RT-qPCR was performed using SYBR^®^ Green Premix Pro Taq HS (AG11733, Accurate Biotechnology, Changsha, China). *GAPDH* served as the internal reference gene, and relative gene expression levels were calculated using the 2^−ΔΔCt^ method [16]. Primer sequences are listed in Table S1.

### 2.9. Western Blot (WB) Analysis

Total cellular protein was extracted using RIPA lysis buffer (P1045, Beyotime, Beijing, China) supplemented with protease and phosphatase inhibitors (P0013B, Beyotime, Beijing, China). Equal amounts of protein were separated by sodium dodecyl sulfate–polyacrylamide gel electrophoresis (SDS-PAGE) and transferred onto polyvinylidene difluoride (PVDF) membranes (E801-02, Vazyme, Nanjing, China). After blocking, membranes were incubated with the primary antibodies overnight at 4 °C, followed by incubation with the corresponding secondary antibodies for 1 h at room temperature. Protein bands were visualized using a chemiluminescence imaging system and quantified by densitometric analysis with ImageJ. GAPDH was used as the internal reference protein [13]. Details of the antibodies are provided in Table S2.

### 2.10. Off-Target Site Detection

Potential off-target sites were predicted using the CCTop platform. The first five candidate sites (Supplementary Table S3) were selected for detection. The genomic fragments of each predicted site were amplified by PCR using specific primers (Supplementary Table S4), and the specificity of the amplified products was verified by 1% agarose gel electrophoresis. Subsequently, the obtained PCR products were subjected to Sanger sequencing to detect the presence of off-target mutations.

### 2.11. Immunofluorescence (IF) Staining

HFSCs were washed three times with phosphate-buffered saline (PBS), fixed with 4% paraformaldehyde at room temperature for 30 min, and permeabilized using permeabilization solution (P0096, Beyotime, Beijing, China) for 1 h. Cells were then blocked with rapid blocking solution (P0269, Beyotime, Beijing, China) for 10 min and incubated with primary antibodies overnight at 4 °C. After washing, cells were incubated with the corresponding fluorescent secondary antibodies for 1 h, followed by nuclear staining with 4′,6-diamidino-2-phenylindole (DAPI) [17]. Fluorescence images were acquired using a fluorescence microscope. Antibody information is provided in Table S5.

### 2.12. Transcriptome Analysis

Total RNA was extracted from sgNC and sgPOSTN using TRIzol reagent (Invitrogen, Waltham, CA, USA). Library preparation and RNA sequencing were performed by LC Bio Technology Co., Ltd. (Hangzhou, China) using the Illumina NovaSeq™ 6000 platform. Raw sequencing data were processed and visualized using the R programming environment (https://www.r-project.org/; accessed on 2 May 2026) and the OmicStudio platform (https://www.omicstudio.cn/tool; accessed on 20 May 2026) [18]. Differentially expressed genes (DEGs) were identified using the DESeq2 package with the thresholds of |fold change| > 2 and *Q* < 0.05. Gene Ontology (GO) and Kyoto Encyclopedia of Genes and Genomes (KEGG) pathway enrichment analyses were then performed.

### 2.13. Metabolome Analysis

Untargeted metabolomic analysis was performed by LC Bio Technology Co., Ltd. (Hangzhou, China) using an ultra-high-performance liquid chromatography–tandem mass spectrometry (UPLC–MS/MS) platform consisting of a Vanquish UPLC system coupled to an Orbitrap Q Exactive™ HF–X mass spectrometer (Thermo Fisher Scientific, Waltham, CA, USA) [19]. Data processing and statistical analyses were conducted in R. Differential metabolites (DMs) were identified using the criteria of |fold change| > 2, *Q* < 0.05, and variable importance in projection (VIP) > 1. Principal component analysis (PCA), orthogonal partial least squares-discriminant analysis (OPLS–DA), and KEGG pathway enrichment analysis were performed to characterize metabolic alterations between experimental groups.

### 2.14. Integrative Analysis of Transcriptome and Metabolome Data

Transcriptomic and metabolomic datasets were integrated using the OmicStudio platform to investigate the relationships between DEGs and DMs. Correlation analyses were performed, and the results were visualized using heatmaps and correlation network diagrams.

### 2.15. Statistical Analysis

Statistical analyses were performed using SPSS version 25.0 (SPSS Inc., Chicago, IL, USA). The Shapiro–Wilk test and Levene’s test were used to assess data normality and homogeneity of variance, respectively. For single-time-point comparisons between two groups (including EdU assay, TUNEL assay, RT-qPCR, and Western blot analysis), an independent two-tailed Student’s *t*-test was applied. For the CCK–8 assay with repeated measurements at multiple time points (0, 24, 48, and 72 h), a two-way repeated-measures analysis of variance (ANOVA) with group as the between-subjects factor and time as the within-subjects factor was used to compare cell viability, followed by Bonferroni post hoc test for pairwise comparisons. Data are presented as mean ± standard error of the mean (SEM). Graphs were generated using GraphPad Prism 8 (GraphPad Software, Inc., San Diego, CA, USA). Differences were considered statistically significant at *p* < 0.05 and highly significant at *p* < 0.01.

## 3. Results

### 3.1. Construction and Validation of the POSTN Editing Vector

**Figure 1:**
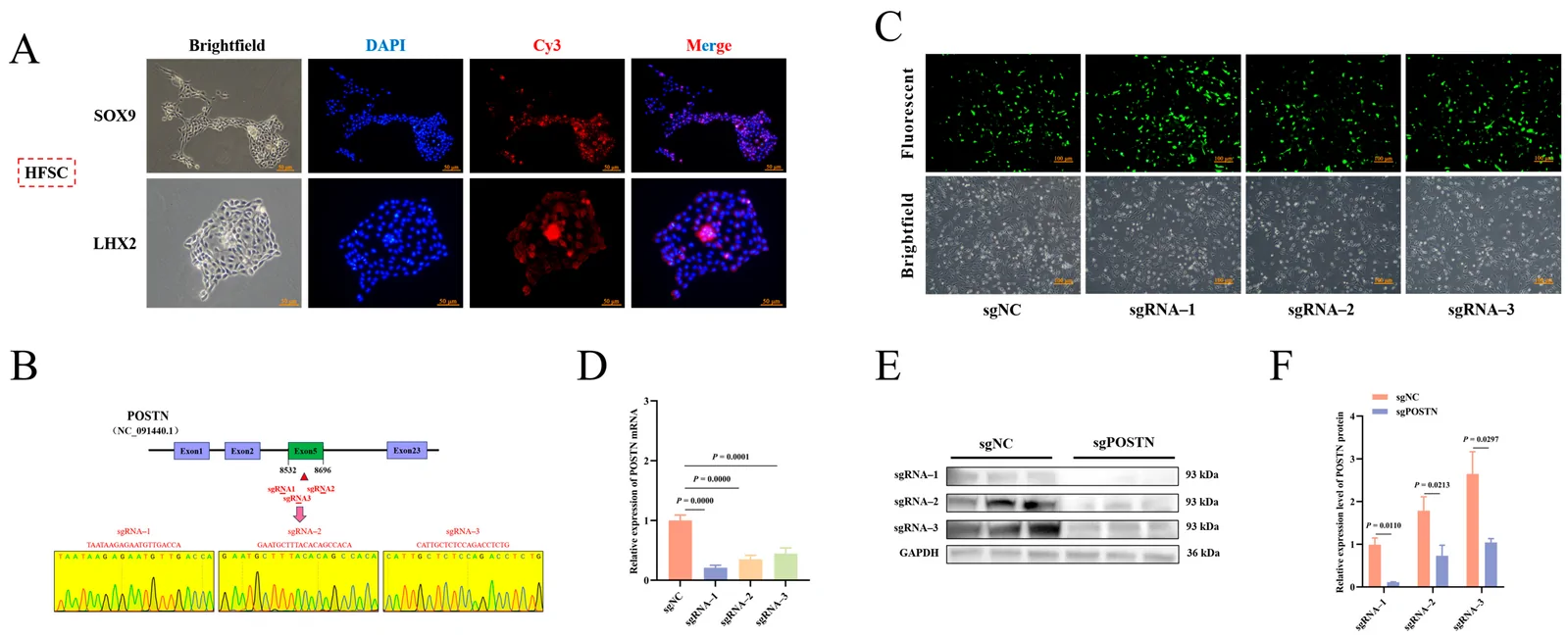
Construction and validation of the POSTN Editing vector. (**A**) IF staining was performed to detect the expression of the HFSC markers SOX9 and LHX2. Scale bar: 50 μm. (**B**) Three sgRNAs targeting exon 5 of *POSTN* were validated by sequencing. (**C**) GFP fluorescence was observed in HFSCs 48 h after transfection. Scale bar: 100 μm. (**D**) The mRNA expression level of *POSTN* was determined by RT-qPCR (*n* = 3). (**E**,**F**) The protein expression level of POSTN was determined by Western blotting (WB) (*n* = 3). Exact *p* values shown; *p* < 0.05 = significant; *p* < 0.01 = highly significant.

Primary HFSCs adhered to the culture surface after isolation and displayed a typical round morphology. Immunofluorescence analysis confirmed stable expression of the HFSC marker proteins SOX9 and LHX2, indicating successful isolation and culture of HFSCs (Figure 1A). Three sgRNAs targeting the *POSTN* gene were cloned into the PX458 vector, and successful plasmid construction was confirmed by Sanger sequencing (Figure 1B). GFP fluorescence was observed 48 h after transfection, demonstrating efficient plasmid delivery into HFSCs (Figure 1C). RT-qPCR and Western blot analyses showed that *POSTN* expression was significantly reduced in all three sgRNA–transfected groups (*p* < 0.05) (Figure 1D–F), with sgRNA–1 exhibiting the highest knockout efficiency.

### 3.2. Validation of POSTN Editing Efficiency in Vitro

**Figure 2:**
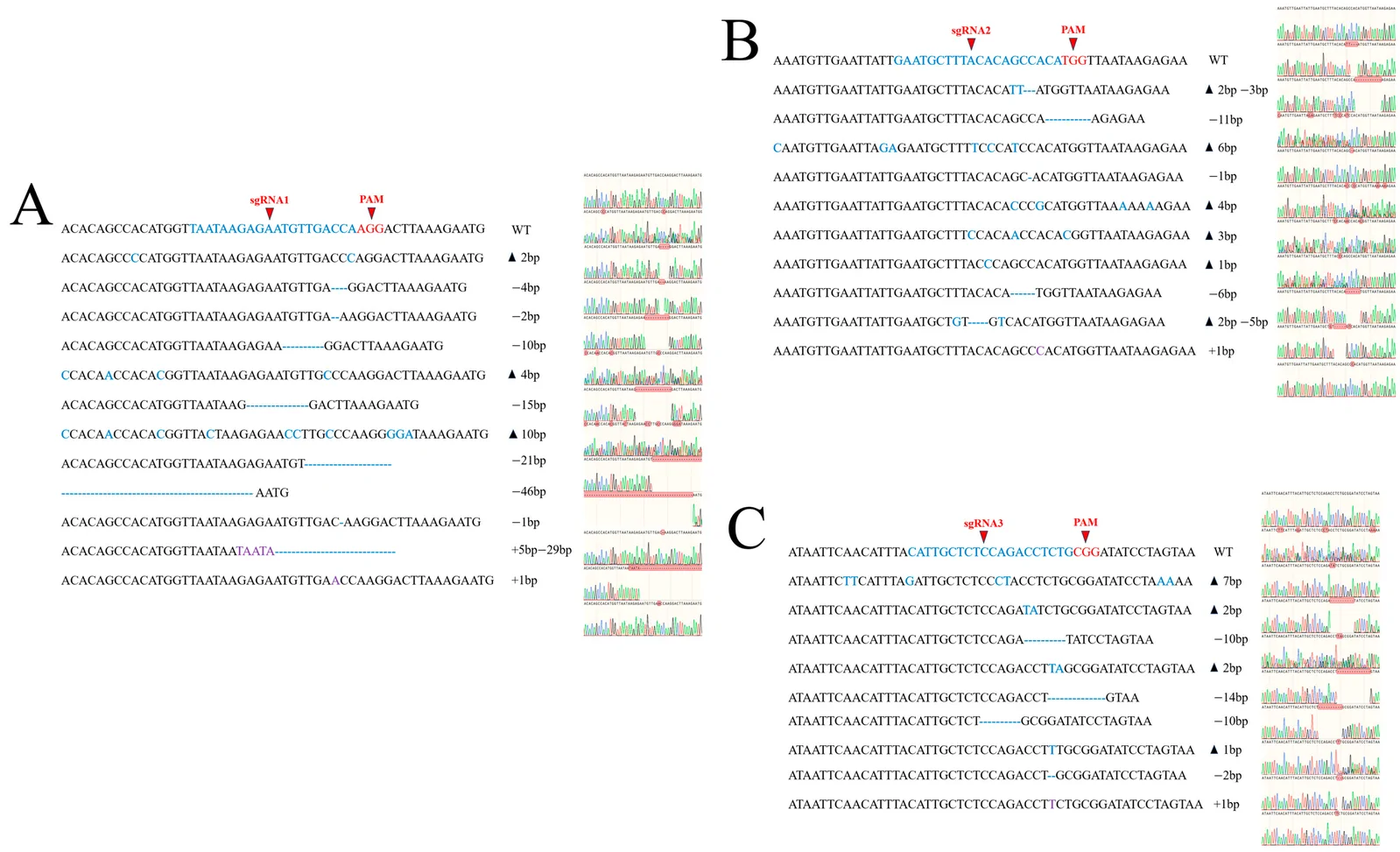
Validation of *POSTN* Editing efficiency in vitro. (**A**) Mutation type produced by sgRNA-1 following targeted editing. (**B**) Mutation type produced by sgRNA-2 following targeted editing. (**C**) Mutation type produced by sgRNA-3 following targeted editing. Blue indicates the sgRNA-binding region, and red indicates the PAM sequence. WT represents the wild-type sequence; “−” indicates base deletion; “+” indicates base insertion; and “▲” indicates base substitution.

TA cloning coupled with Sanger sequencing was performed to evaluate genome-editing efficiency. Fifteen independent colonies were randomly selected for sequencing for each sgRNA. The editing efficiencies of sgRNA-1, sgRNA-2, and sgRNA-3 were 80.0% (12/15), 66.7% (10/15), and 60.0% (9/15), respectively. Multiple insertion and deletion mutation types were identified at the target site (Figure 2A–C). Based on its better editing efficiency, sgRNA–1 was selected for subsequent experiments.

### 3.3. Detection of Off-Target Effect of POSTN Editing Vector

**Figure 3:**
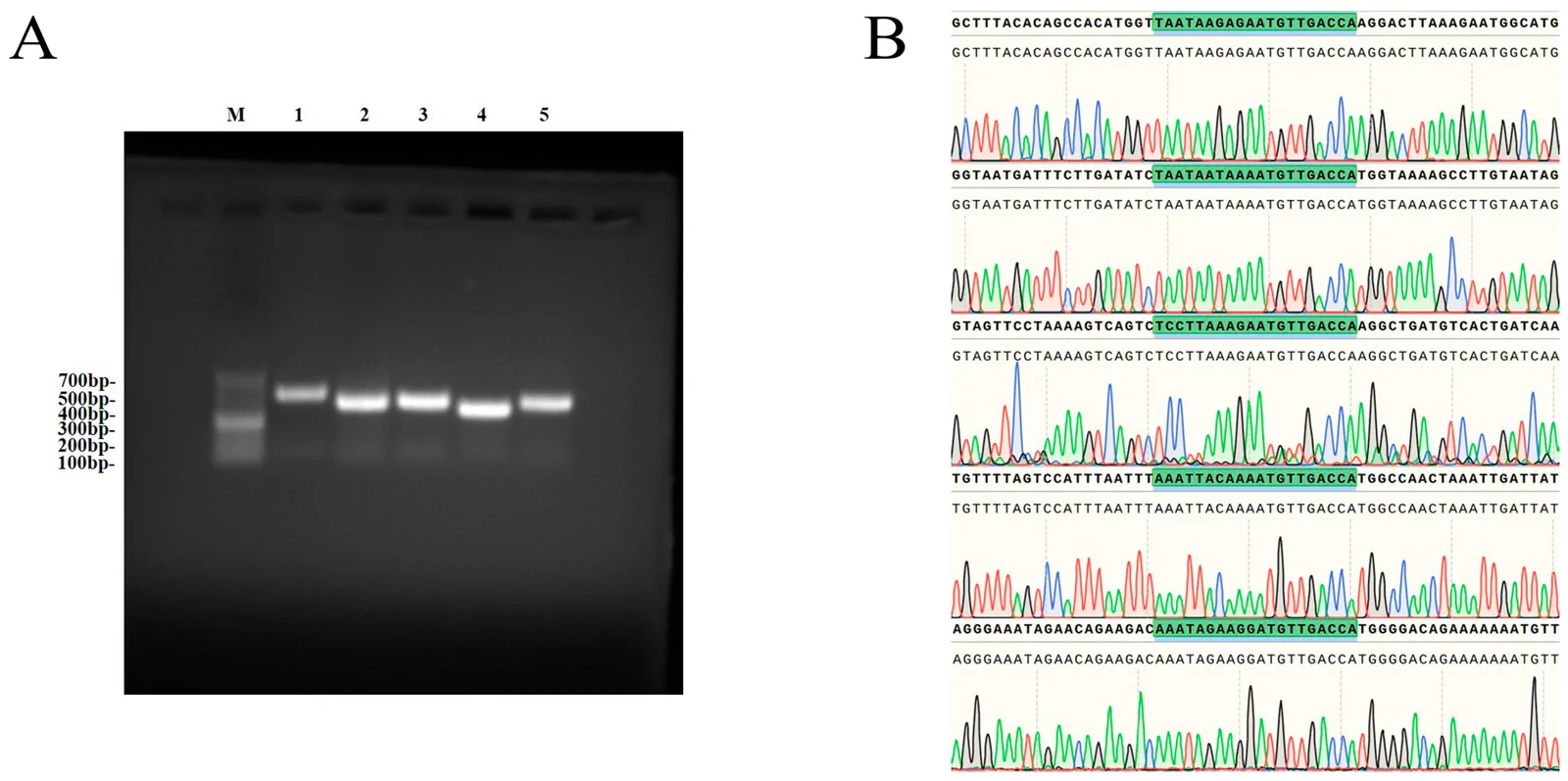
Detection of off-target effect of *POSTN* editing vector. (**A**) The PCR products of 5 candidate off-target sites were identified by 1% agarose gel electrophoresis. M: DL1000 DNA marker; lanes 1–5 were the amplification products of 5 predicted off-target sites. (**B**) Sanger sequencing verified the mutation of 5 predicted off-target sites, and the green highlighted region was the putative off-target region.

In order to verify the potential off-target risk of the sgRNA1 editing vector, five candidate off-target sites were amplified by PCR. Agarose gel electrophoresis confirmed that the size of the amplified product was consistent with the expected, and the primer specificity was good (Figure 3A). After Sanger sequencing of the purified product, no mutation was detected at all candidate sites (Figure 3B). The results showed that the editing vector did not show off-target effects at the analyzed sites.

### 3.4. POSTN Editing Inhibits Proliferation and Promotes Apoptosis of HFSCs

**Figure 4:**
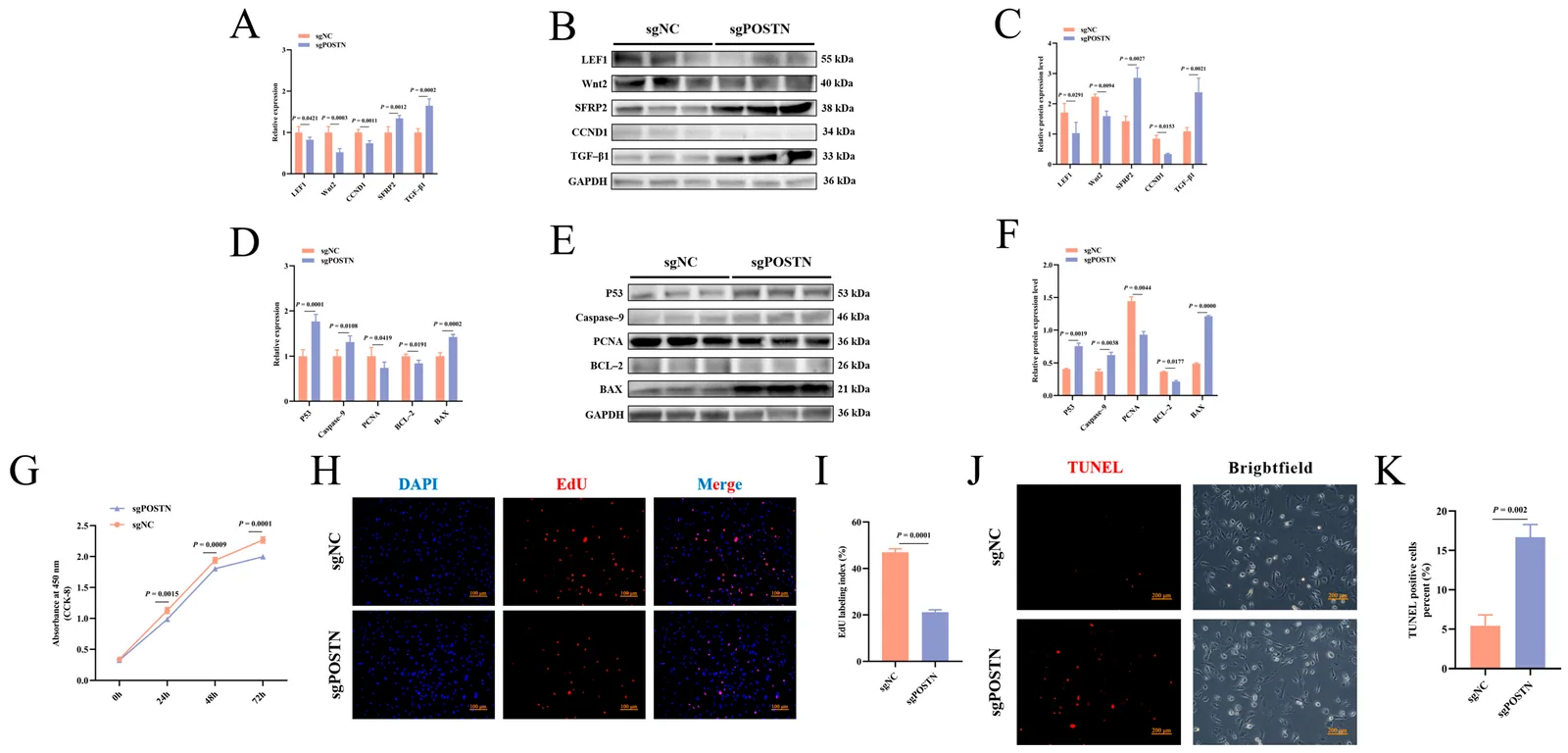
*POSTN* editing inhibits proliferation and promotes apoptosis of HFSCs. (**A**) The expression levels of HF development-related genes were determined by RT-qPCR (*n* = 3). (**B**,**C**) The protein expression levels of HF development-related genes were determined by WB (*n* = 3). (**D**) The expression levels of genes associated with cell proliferation and apoptosis were determined by RT-qPCR (*n* = 3). (**E**,**F**) The protein expression levels of cell proliferation- and apoptosis-related genes were determined by WB (*n* = 3). (**G**) Cell proliferation was assessed using the CCK–8 assay at 0, 24, 48, and 72 h after *POSTN* knockout (*n* = 3). (**H**,**I**) Cell proliferation was evaluated by EdU staining. Scale bar: 100 μm. (**J**,**K**) Apoptosis was assessed by TUNEL staining. Scale bar: 200 μm. Exact *p* values shown; *p* < 0.05 = significant; *p* < 0.01 = highly significant.

HF morphogenesis and cyclic regeneration depend on the activation and functional maintenance of HFSCs. Following *POSTN* editing, the expression levels of the HF development-related genes *LEF1*, *CCND1*, and *Wnt2* were significantly decreased, whereas the expression of *SFRP2* and *TGF*–*β1* was significantly increased (*p* < 0.05) (Figure 4A–C). Regarding cell proliferation and apoptosis, *POSTN* editing significantly increased the expression of the pro-apoptotic genes *Bax*, *Caspase*–*9*, and *P53*, while reducing the expression of the anti-apoptotic gene *BCL*–*2* and the proliferation marker *PCNA* (*p* < 0.05) (Figure 4D–F). Consistent with these molecular changes, CCK–8, EdU, and TUNEL assays demonstrated that *POSTN* editing significantly suppressed HFSCs’ proliferation and promoted apoptosis (*p* < 0.05) (Figure 4G–K). In summary, these findings indicate that POSTN can positively regulate the proliferative and apoptotic activity of HFSCs, and contribute to maintaining the proliferation–apoptosis homeostasis of HFSCs.

### 3.5. POSTN Editing Induced Changes in Transcriptional Expression and Functional Pathway Response of HFSCs

**Figure 5:**
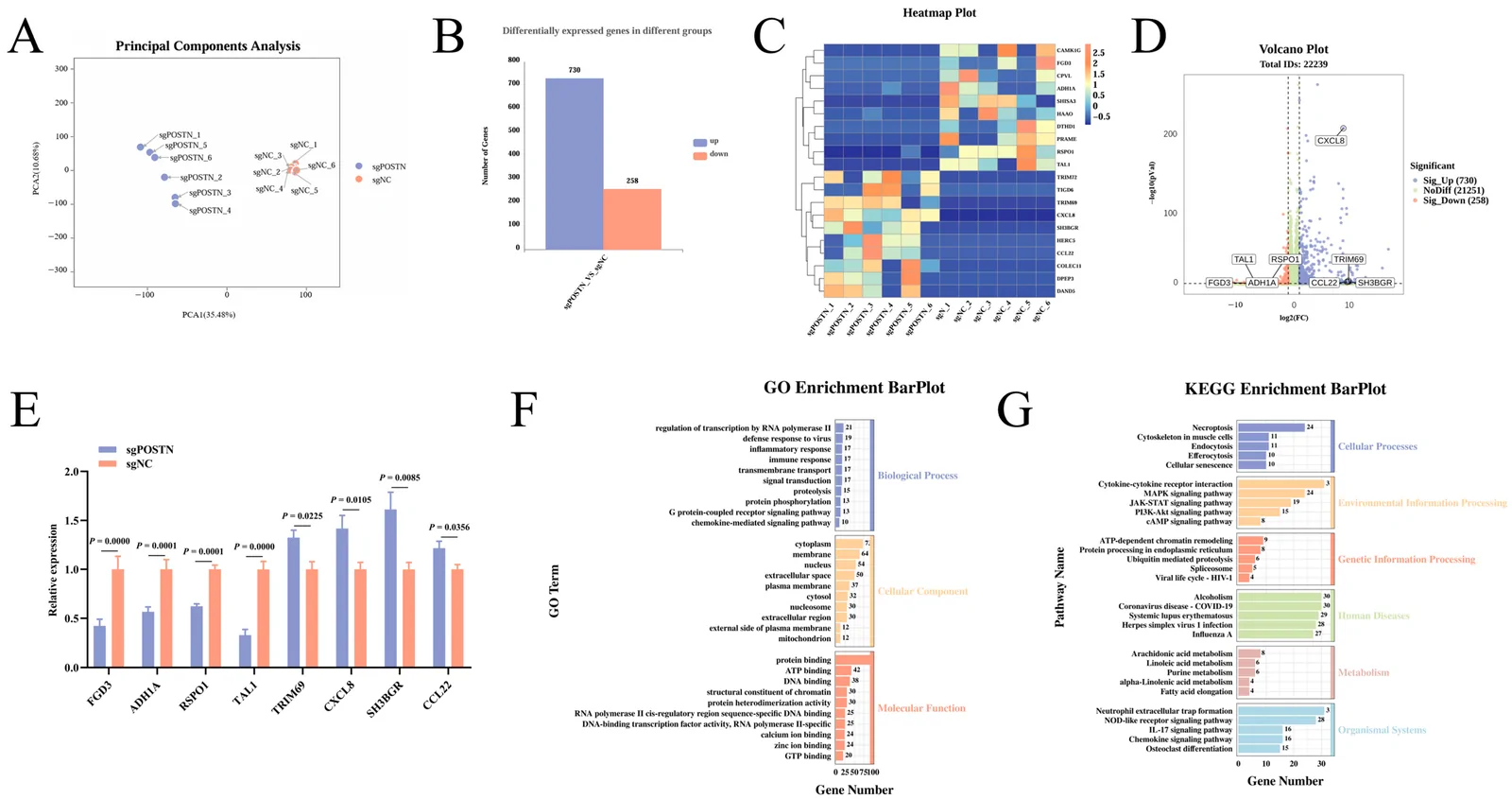
*POSTN* Editing alters the transcriptomic profile and functional pathways of HFSCs. (**A**) PCA showing the separation between the sgNC and sgPOSTN. (**B**) Overall distribution of DEGs between the two groups. (**C**) Heat map showing the 10 most significantly upregulated and 10 most significantly downregulated genes. (**D**) Volcano plot showing the overall distribution of DEGs between the sgNC and sgPOSTN. (**E**) Representative DEGs were validated by RT–qPCR (*n* = 3). (**F**) The 10 most significantly enriched GO functional categories. (**G**) The five most significantly enriched KEGG signaling and metabolic pathways. Exact *p* values shown; *p* < 0.05 = significant; *p* < 0.01 = highly significant.

To investigate the transcriptional changes induced by *POSTN* editing, RNA sequencing was performed using sgNC and sgPOSTN. A total of 244,011,604 and 168,961,934 raw reads were obtained from the sgNC and sgPOSTN, respectively. High-quality reads with Q30 > 98% were retained for subsequent analyses (Table S6). PCA demonstrated clear separation between the two groups (Figure 5A). A total of 988 DEGs were identified, including 730 upregulated and 258 downregulated genes (Figure 5B). Heatmap analysis displayed the 10 most significantly upregulated and downregulated genes (Figure 5C). Furthermore, eight genes associated with HF development were validated by RT–qPCR, and their expression patterns were consistent with the RNA-seq results (Figure 5D,E). GO enrichment analysis indicated that the identified DEGs were primarily associated with immune and inflammatory responses, signal transduction, cytoplasmic and membrane components, and molecular functions including protein binding and ATP binding (Figure 5F). KEGG pathway analysis further demonstrated significant enrichment of several signaling pathways, including the MAPK, PI3K–Akt, and cAMP pathways (Figure 5G). These findings demonstrate that *POSTN* editing significantly remodels the transcriptomic profile of HFSCs and alters multiple biological processes and signaling pathways.

### 3.6. POSTN Editing Alters the Metabolomic Profile of HFSCs

**Figure 6:**
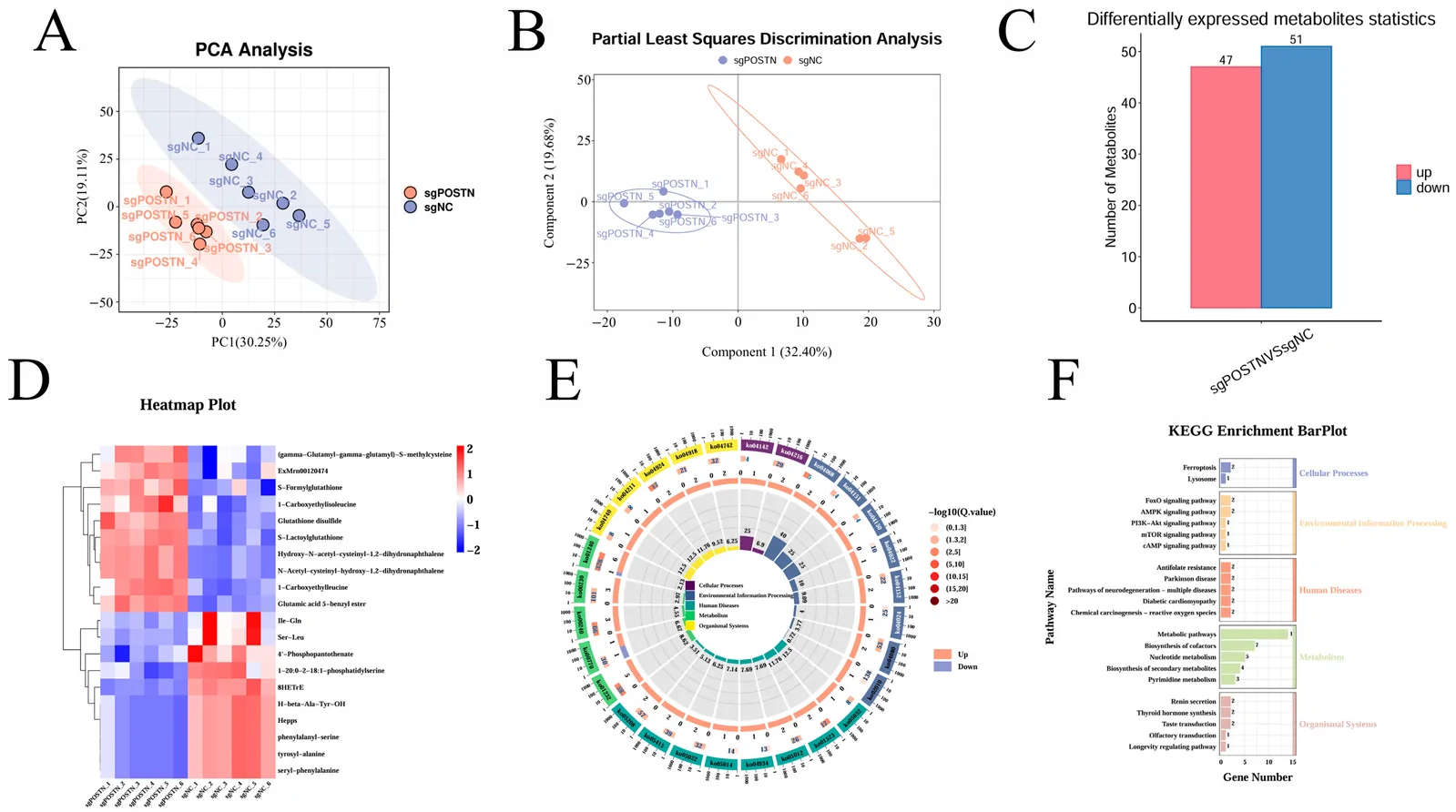
*POSTN* editing induces metabolic changes in HFSCs. (**A**) PCA showing the distribution of samples in each group. (**B**) OPLS-DA score plot. (**C**) Histogram showing the number of DMs. (**D**) Heat map showing the 10 most significantly upregulated and 10 most significantly downregulated metabolites. (**E**) KEGG pathway enrichment bubble plot of DMs. (**F**) The five most significantly enriched KEGG signaling and metabolic pathways.

To investigate the metabolic alterations induced by *POSTN* editing, untargeted metabolomic analysis was performed on sgNC and sgPOSTN using UPLC–MS/MS. PCA demonstrated clear separation between the sgNC and sgPOSTN (Figure 6A), while OPLS-DA further confirmed distinct metabolic profiles between the two groups (Figure 6B). A total of 98 DMs were identified, including 47 upregulated and 51 downregulated metabolites (Figure 6C). Among the upregulated metabolites, S-formylglutathione, glutathione disulfide, and S–lactoylglutathione showed significant accumulation, whereas phenylalanyl-serine, tyrosyl-alanine, and seryl-phenylalanine were significantly downregulated in *POSTN*-edited cells (Figure 6D). KEGG pathway enrichment analysis revealed that the DMs were primarily enriched in biosynthesis of cofactors, nucleotide metabolism, and several signaling pathways, including the FoxO, AMPK, and cAMP pathways (Figure 6E,F). These findings indicate that *POSTN* editing significantly alters the metabolic landscape of HFSCs.

### 3.7. Integrative Analysis of Transcriptomic and Metabolomic Data

**Figure 7:**
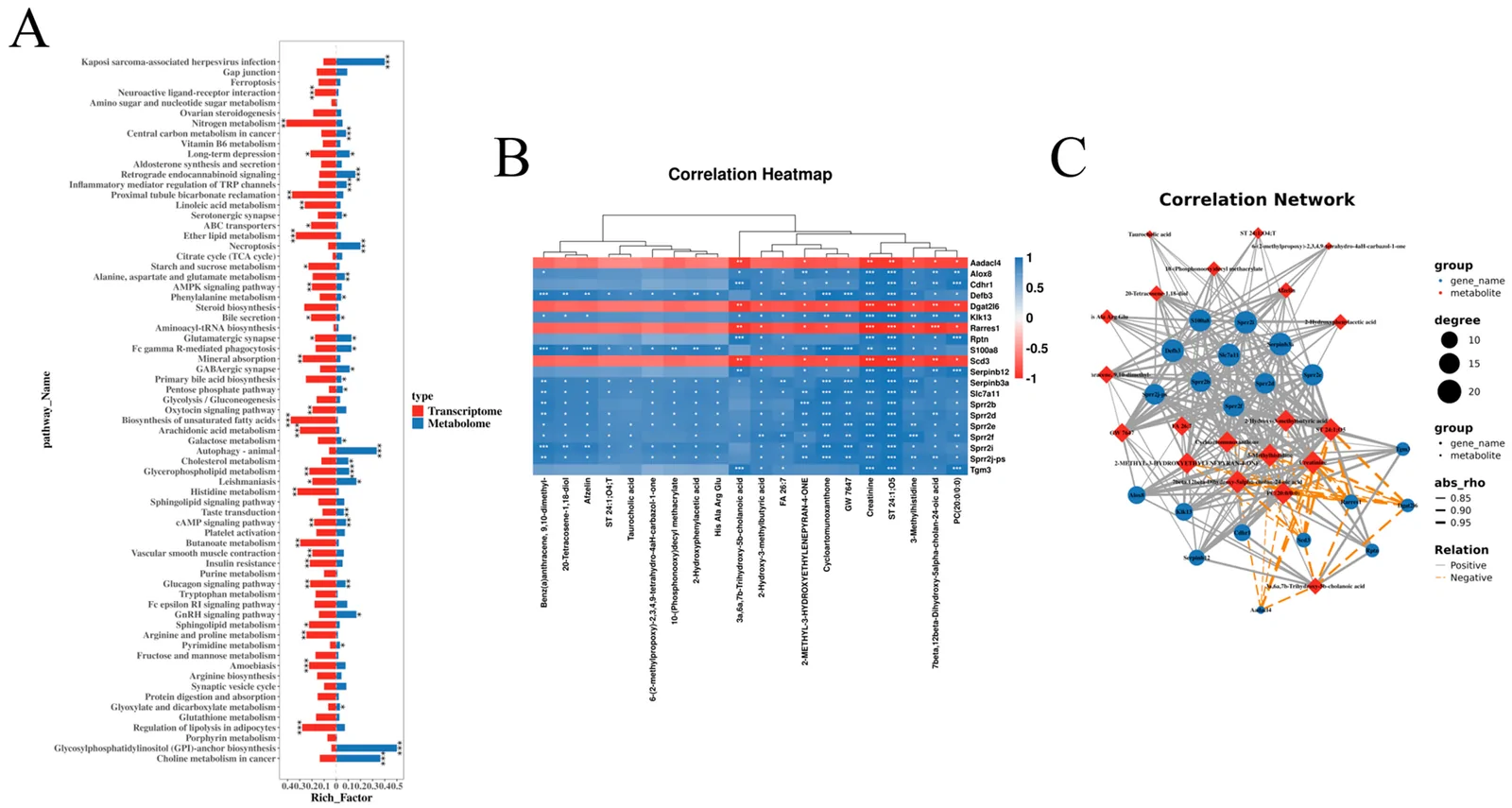
Integrative Analysis of Transcriptomic and Metabolomic Data. (**A**) KEGG enrichment analysis showed the enrichment of DEGs and DMs. The abscissa is the enrichment factor, and the ordinate is the pathway name. (**B**) The correlation heat map of DEGs and DMs, and the color gradient reflect the strength and direction of the correlation. (**C**) The interaction network shows the relationship between DEGs and DMs. The node size represents the degree of significant difference.

To comprehensively investigate the molecular changes associated with *POSTN* editing, transcriptomic and metabolomic datasets were integrated for correlation analysis. The combined analysis revealed significant enrichment of differential genes and metabolites across several biological pathways, including glycerophospholipid metabolism, glucagon signaling, and cAMP signaling (Figure 7A). Pearson correlation analysis was then performed to investigate associations between DEGs and DMs. The heatmap illustrates the top 20 gene–metabolite correlations (Figure 7B), while the correlation network visualizes the interactions between DEGs and DMs (Figure 7C). These results suggest coordinated transcriptomic and metabolomic alterations in HFSCs following *POSTN* editing.

### 3.8. cAMP/PKA/CREB Is a Key Signaling Pathway for Transcription and Metabolism

**Figure 8:**
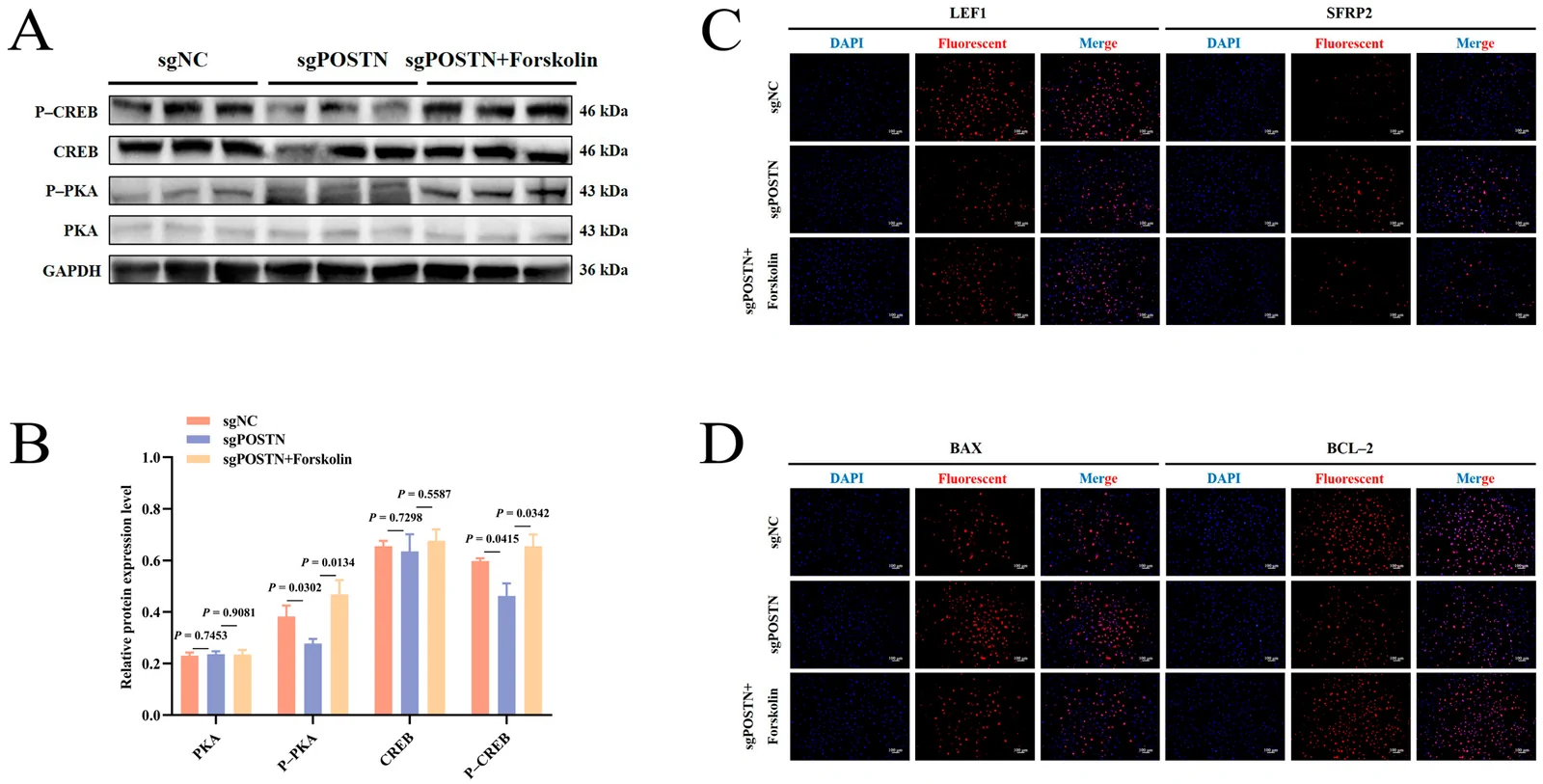
cAMP/PKA/CREB is a key signaling pathway for transcription and metabolism. (**A**,**B**) WB was used to analyze the key proteins in the cAMP/PKA/CREB pathway and their expression changes after Forskolin treatment (*n* = 3). (**C**,**D**) The expression of *LEF1*, *SFRP2*, *BAX* and *BCL–2* and their changes after Forskolin treatment were analyzed by IF. Scale bar: 100 μm. Exact *p* values shown; *p* < 0.05 = significant; *p* < 0.01 = highly significant.

The cAMP pathway is a key signaling pathway that regulates cell proliferation, apoptosis and gene transcription. Multi-omics analysis showed that the cAMP signaling pathway was the core pathway of DEGs’ and DMs’ co-enrichment, suggesting that it played a key role in *POSTN*-mediated HFSC functional regulation. The results of WB showed that *POSTN* editing could significantly down-regulate the phosphorylation levels of PKA and CREB, the downstream effectors of the cAMP pathway (*p* < 0.05) (Figure 8A,B). After intervention with the cAMP pathway-specific agonist Forskolin in *POSTN*-edited HFSCs, the protein expression levels of P-PKA and P-CREB were significantly increased (*P* < 0.05) (Figure 8A,B), and the pathway inhibition effect mediated by *POSTN* editing was effectively reversed.

IF results further showed that after *POSTN* editing, the fluorescence intensity of the HF development-related molecule *LEF1* was significantly reduced, and the fluorescence intensity of the HF development inhibitor *SFRP2* was increased (Figure 8C,D). At the same time, the fluorescence intensity of the pro-apoptotic protein *BAX* was enhanced, and the fluorescence intensity of the anti-apoptotic protein *BCL*-*2* was weakened (Figure 8C,D). Forskolin treatment can effectively reverse the expression of the above molecules (Figure 8C,D). These results indicate that POSTN mediates the proliferation and apoptosis of HFSCs by regulating the activation level of the cAMP/PKA/CREB signaling pathway.

## 4. Discussion

HF regeneration is a cyclic process consisting of the anagen, catagen, and telogen phases [1,13]. HFSCs, which reside in the bulge region of the hair follicle, are a type of multipotent stem cell with self-renewal capacity and multilineage differentiation potential. Periodic activation and quiescence of HFSCs are essential for maintaining HF homeostasis and supporting cyclic hair regeneration [20,21,22]. The biological functions of HFSCs are regulated by the ECM niche and multiple intracellular signaling pathways that collectively coordinate cell proliferation, apoptosis, and differentiation [23]. As a matricellular protein abundantly expressed in the ECM, POSTN participates in various biological processes, including tissue remodeling, wound repair, and regulation of cell proliferation and differentiation. In this study, a *POSTN*-edited cell model was established in primary HFSCs using CRISPR/Cas9–mediated genome editing to investigate the role of POSTN in HFSCs’ biology. Functional assays combined with transcriptomic and metabolomic analyses demonstrated that *POSTN* editing suppressed HFSCs’ proliferation, promoted apoptosis, and induced extensive molecular changes, indicating that POSTN is required for maintaining HFSCs’ homeostasis.

POSTN has been reported to interact with integrins and other extracellular components, facilitating communication between the extracellular matrix and intracellular signaling pathways involved in cell growth, survival, and differentiation [24]. Previous studies have shown that POSTN promotes wound healing by promoting myofibroblast proliferation and dermal fibroblast activation [9,25,26]. Furthermore, *POSTN* editing suppresses osteogenic differentiation of dental pulp stem cells, promotes adipogenic differentiation, and decreases their proliferative capacity [27]. These findings support a broad role for POSTN in regulating stem cell fate and tissue regeneration. Consistent with these findings, *POSTN* editing in our study significantly decreased the expression of the HF development-related genes *LEF1*, *CCND1*, and *Wnt2*, while increasing the expression of the inhibitory factors *SFRP2* and *TGF*–*β1*. Moreover, *POSTN* editing increased the expression of the pro-apoptotic genes *Bax*, *Caspase*–*9*, and *P53* and reduced the expression of the anti-apoptotic gene *BCL*–*2* and the proliferation marker *PCNA*. Functional assays further confirmed that *POSTN* editing inhibited HFSCs’ proliferation and promoted apoptosis. These results suggest that POSTN contributes to the maintenance of the balance between proliferation and apoptosis in HFSCs, supporting normal HF development.

Transcriptomic analysis provided further insight into the molecular mechanisms underlying the effects of *POSTN* editing. RNA sequencing identified 988 DEGs, and enrichment analyses indicated that these genes were primarily associated with immune and inflammatory responses, signal transduction, and multiple signaling pathways, including the MAPK, PI3K–Akt, and cAMP pathways. Among them, the MAPK pathway is a major mediator of extracellular signal transduction and plays important roles in regulating cell proliferation, differentiation, stress responses, and HF development [28,29]. Similarly, activation of the PI3K–Akt pathway has been reported to promote HFSCs’ proliferation [30,31] and facilitate the transition of HFs from the telogen phase to the anagen phase [32,33]. The cAMP signaling pathway regulates stem cell self-renewal and differentiation through protein kinase A (PKA)-dependent signaling cascades [34].

Metabolomic analysis further complemented the molecular profiling of POSTN function and revealed potential links between POSTN and cellular metabolic homeostasis in HFSCs. *POSTN* editing significantly altered the metabolomic profile of HFSCs, leading to the identification of 98 DMs. These metabolites were primarily enriched in pathways related to cofactor biosynthesis, nucleotide metabolism, and several signaling pathways, including the FoxO, AMPK, and cAMP pathways. Nucleotide metabolism and cofactor biosynthesis provide essential substrates for macromolecule synthesis, energy production, and cell proliferation. Disruption of these metabolic processes has been associated with reduced stem cell proliferation and impaired self-renewal capacity [35,36]. The AMPK–FoxO signaling axis plays a key role in coordinating cellular responses to metabolic stress by regulating cell cycle progression, apoptosis, and energy homeostasis. Abnormal over-activation can further aggravate cell metabolic disorders and functional damage [37,38,39]. In addition, the activation of the cAMP pathway can drive the glycolysis metabolic remodeling of HFSCs, which is the necessary molecular basis for the activation of HFSCs and the promotion of HF growth [35].

Integrated transcriptomic and metabolomic analyses provided further insight into the molecular basis of POSTN-mediated regulation of HFSCs’ homeostasis. Correlation analysis demonstrated coordinated alterations between DEGs and DMs, indicating interactions between transcriptional and metabolic processes following *POSTN* editing. It is worth noting that the cAMP signaling pathway is significantly enriched in both transcriptional and metabolic analyses, suggesting that it may play a regulatory role in *POSTN* editing-mediated biological effects. As a key second messenger in cells, cAMP is widely involved in regulating cell proliferation, differentiation and metabolic homeostasis. It activates protein kinase A (PKA), which in turn phosphorylates the cAMP-response element binding (CREB) protein at Serine^133^. Phosphorylated CREB specifically binds to CRE in the promoter regions of target genes, and drives the transcriptional expression of genes related to proliferation and differentiation [40,41,42]. To further clarify the regulatory relationship between POSTN and the cAMP pathway, we detected the phosphorylation levels of core pathway proteins via WB. The results showed that *POSTN* editing significantly reduced the phosphorylation levels of PKA and its downstream effector CREB, confirming that *POSTN* editing inhibits the activity of the cAMP/PKA/CREB signaling pathway. Upon exogenous treatment with forskolin, a specific cAMP pathway agonist, the downregulated phosphorylation levels of PKA and CREB were significantly restored, accompanied by upregulated expression of the anti-apoptotic gene *BCL*–*2* and the HF development-related gene *LEF1*. Collectively, these results indicate that *POSTN* editing modulates the functional status and metabolic homeostasis of HFSCs at least partially by inhibiting the activity of the cAMP/PKA/CREB signaling pathway.

In summary, this study revealed the transcriptional and metabolic changes caused by *POSTN* editing through multi-omics analysis, and screened out several candidate regulatory networks related to HFSCs’ function. The cAMP/PKA/CREB pathway is the core functional pathway that mediates POSTN regulation of HFSCs’ proliferation and apoptosis. These findings suggest that POSTN maintains HFSCs’ homeostasis by coordinating cell transcription and metabolic networks, and is involved in the functional regulation of HFSCs at least in part through the cAMP/PKA/CREB pathway.

At the same time, this study still has some limitations. First of all, functional experiments and multi-omics analysis were carried out in HFSCs cultured in vitro, which could not completely restore the complex extracellular matrix microenvironment and periodic regulation mechanism of HF in vivo. The regulation of POSTN on HFSCs still needs to be verified in the hair growth model of living animals. Secondly, among the multiple pathways enriched by omics, only the cAMP/PKA/CREB signaling axis has completed preliminary verification, and the regulatory effects of other pathways such as MAPK and PI3K-Akt are still candidate mechanisms, which need to be further confirmed by subsequent targeted experiments. Thirdly, this study only used the CRISPR/Cas9-edited loss-of-function strategy, and the subsequent complementary functions were experimentally and systematically verified to further clarify the relationship between POSTN and HFSCs’ functional homeostasis.

## 5. Conclusions

**Figure 9:**
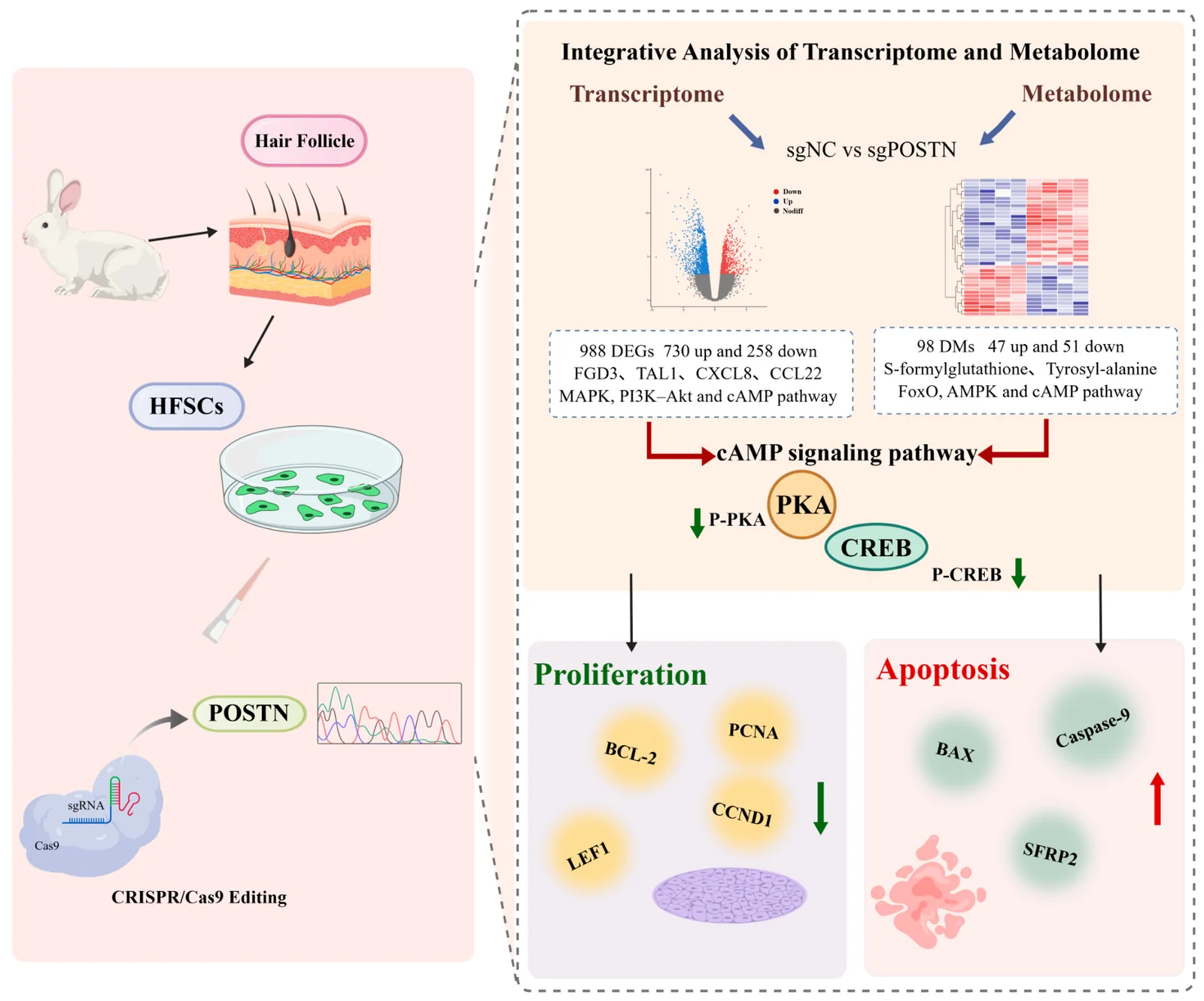
The molecular mechanism pattern of *POSTN* editing regulating the proliferation and apoptosis of HFSCs.

This study systematically investigated the biological role of POSTN in HFSCs using CRISPR/Cas9-mediated gene editing in combination with transcriptomic and metabolomic analyses. *POSTN* editing inhibited HFSCs’ proliferation, promoted apoptosis, and induced coordinated transcriptomic and metabolomic alterations. Integrated multi-omics analysis uncovered extensive correlations between DEGs and DMs, and highlighted the cAMP pathway as the core regulatory axis mediating POSTN function in HFSCs. WB validation further confirmed that POSTN editing reduces PKA and CREB phosphorylation, indicating that POSTN regulates HFSC proliferation and apoptosis partially via the cAMP/PKA/CREB signaling cascade. These findings expand the current understanding of the molecular mechanism of POSTN in HF biology and provide a theoretical foundation for future mechanistic studies and the development of therapeutic strategies targeting HF regeneration and related disorders (Figure 9).

## Figures and Tables

**Table 1:** Sequences of sgRNA targeting sites.

Name	Sequence of sgRNA	PAM
sgRNA–1	TAATAAGAGAATGTTGACCA	AGG
sgRNA–2	GAATGCTTTACACAGCCACA	TGG
sgRNA–3	CATTGCTCTCCAGACCTCTG	CGG

**Table 2:** Primers for the amplification of the target fragments.

Name	Primer Sequences (5′ to 3′)
sgRNA1–F	ggatcttccagagatTCTGGAAATGTCCAAGGAGTATCTG
sgRNA1–R	ctgccgttcgacgatGGTAATGGTAATTGTACTTATGTTATACTCAA
sgRNA2–F	ggatcttccagagatCCATGATTTACATCAATATTGTCTCTTC
sgRNA2–R	ctgccgttcgacgatACTAAATTATTTGGTAAAAAAAACAGTAACTAG
sgRNA3–F	ggatcttccagagatTCTGGAAATGTCCAAGGAGTATCTG
sgRNA3–R	ctgccgttcgacgatTGTTATATAAAAAAATTACTTAAAACACACCA

**Table 3:** TA cloning vector M13 universal sequencing primers.

Name	Primer Sequences (5′ to 3′)
M13–F	TGTAAAACGACGGCCAGT
M13–R	CAGGAAACAGCTATGACC

## Data Availability

All data generated or analyzed during this study are included in this published article and its Supplementary Information Files.

## Supplementary Materials

The following supporting information can be downloaded at: https://www.mdpi.com/article/10.3390/cells15171516/s1, Table S1: Primer sequences used for RT-qPCR. Table S2: The antibodies information. Table S3: Potential off-target sites for sgRNA1. Table S4: Primers of off-target sites. Table S5: The antibodies information. Table S6: Overview of transcriptome sequencing data. Supplementary File S1: WB original image and supplementary experiment.
