# Supplementary material for: CRISPR-Mediated POSTN Editing Modulates Proliferation, Apoptosis, and Molecular Profiles of Primary Rabbit Hair Follicle Stem Cells via the cAMP/PKA/CREB Signaling Pathway
Source: Cells. 2026 Aug 23;15(17):1516. doi: 10.3390/cells15171516 (PMC13564656; doi:10.3390/cells15171516)
Supplement: Supplementary file 1 [file cells-15-01516-s001.zip › Supplementary File S1. WB original image and supplementary experiment.pdf]

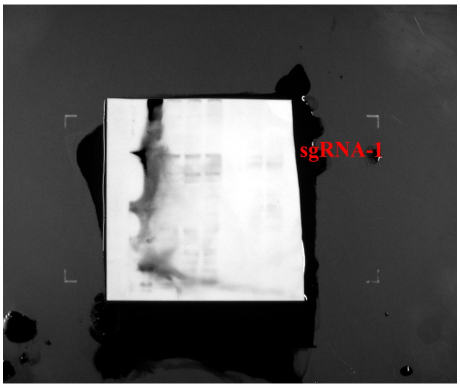

Fig S1E The protein expression of sgRNA–1.

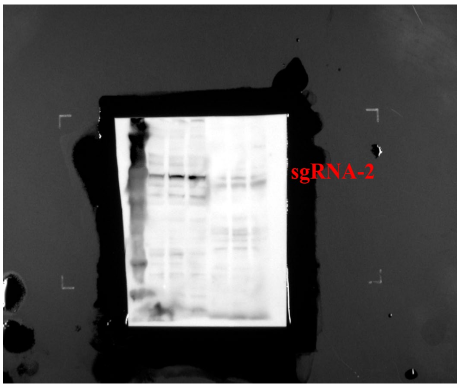

Fig S1E The protein expression of sgRNA–2.

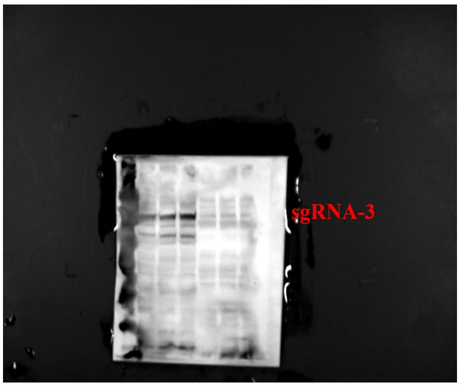

Fig S1E The protein expression of sgRNA–3.

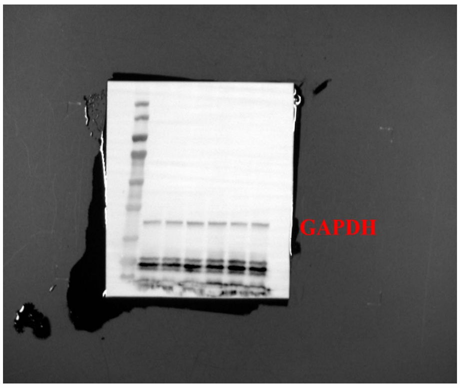

Fig S1E The protein expression of GAPDH.

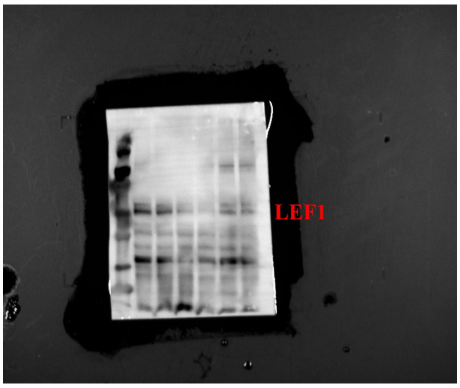

Fig S4B The protein expression of LEF1.

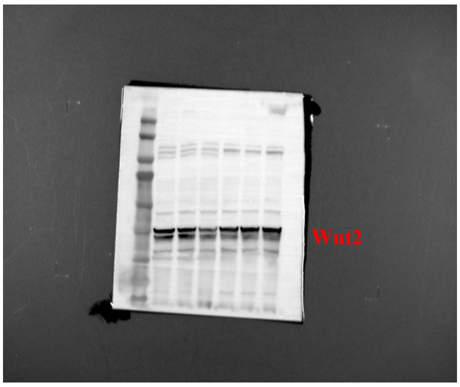

Fig S4B The protein expression of Wnt2.

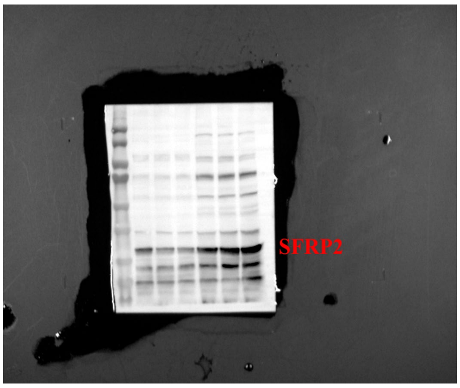

Fig S4B The protein expression of SFRP2.

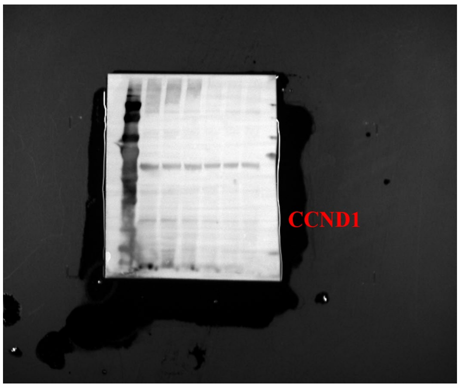

Fig S4B The protein expression of CCND1.

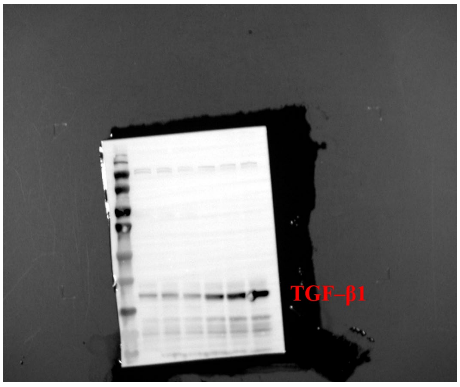

Fig S4B The protein expression of TGF-β1.

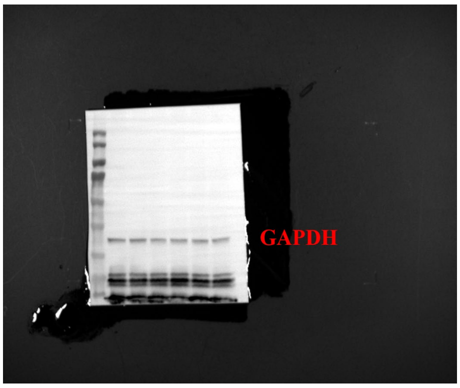

Fig S4B The protein expression of GAPDH.

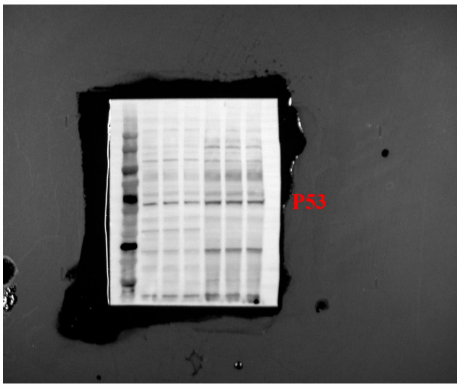

Fig S4E The protein expression of P53.

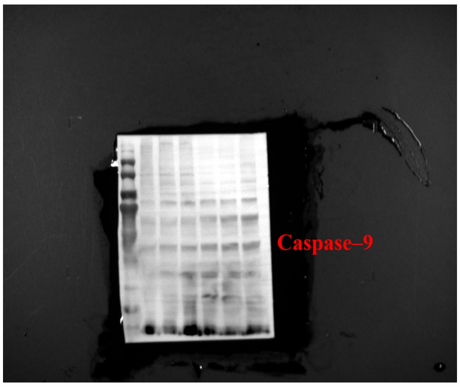

Fig S4E The protein expression of Caspase–9.

.

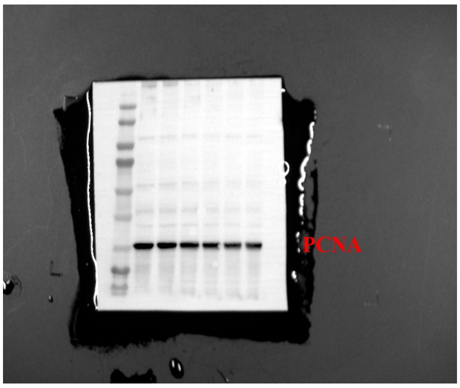

Fig S4E The protein expression of PCNA.

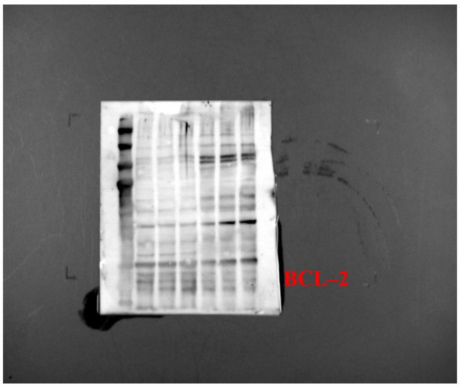

Fig S4E The protein expression of BCL–2.

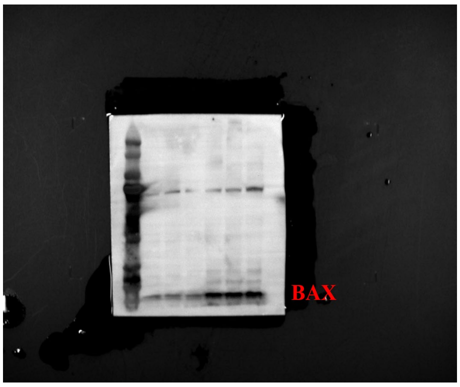

Fig S4E The protein expression of BAX.

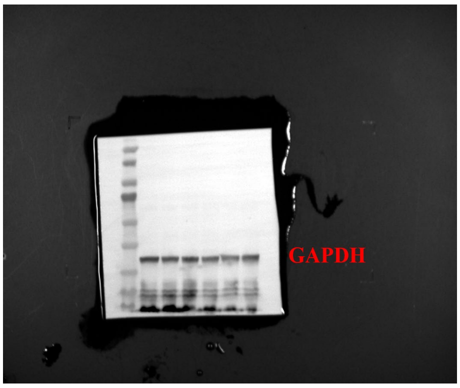

Fig S4E The protein expression of GAPDH.

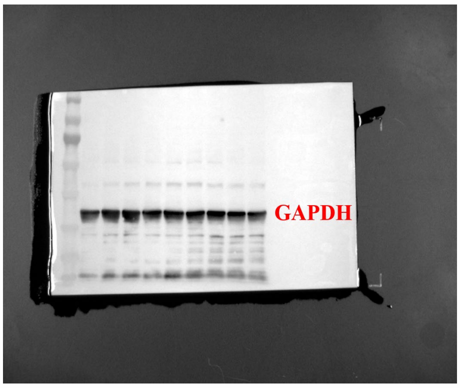

Fig S8A The protein expression of GAPDH.

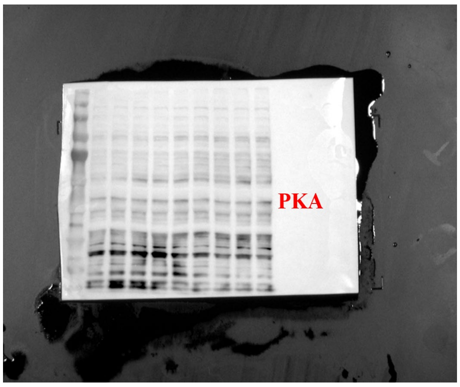

Fig S8A The protein expression of PKA.

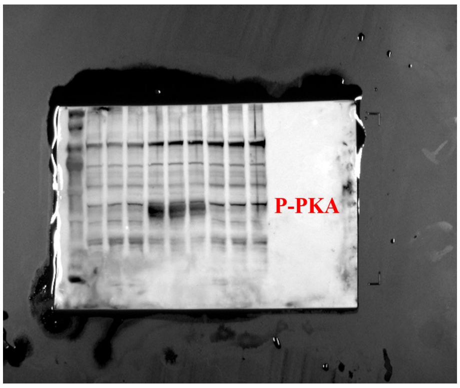

Fig S8A The protein expression of P-PKA.

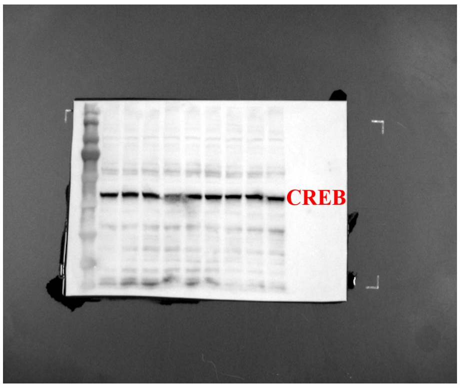

Fig S8A The protein expression of CREB.

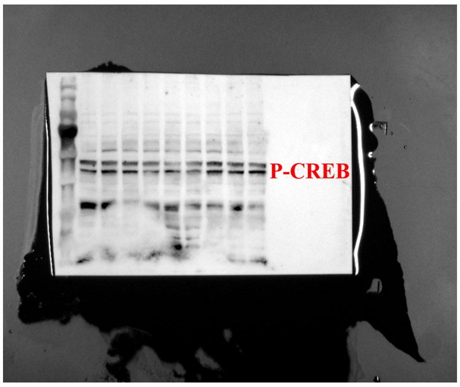

Fig S8A The protein expression of P-CREB.

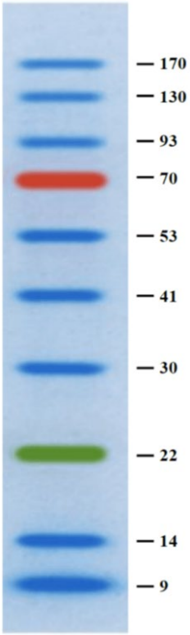

Marker of WB

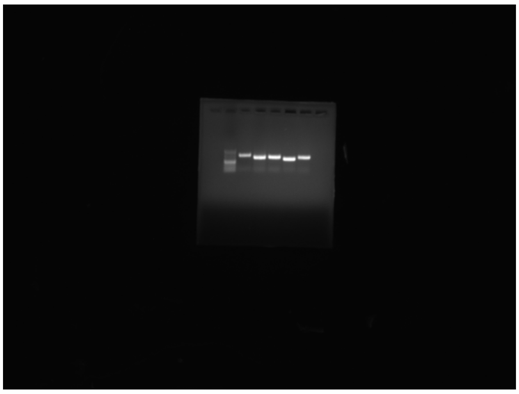

Fig S3A Analysis of five predicted potential off-target sites by 1% agarose gel electrophoresis.

Hematoxylin eosin (HE) Staining

Part of the skin tissue was selected and treated with paraformaldehyde (BL539A; Biosharp, Hefei, China) was fixed for 48h, dehydrated, embedded in paraffin, sliced, dewaxed, hydrated with ethanol, and washed with distilled water. The sections were stained with hematoxylin for 8 min, and then stained with eosin for 1 min. After that, the sections were treated with xylene for permeabilization, sealed with neutral glue, and observed under an inverted microscope.

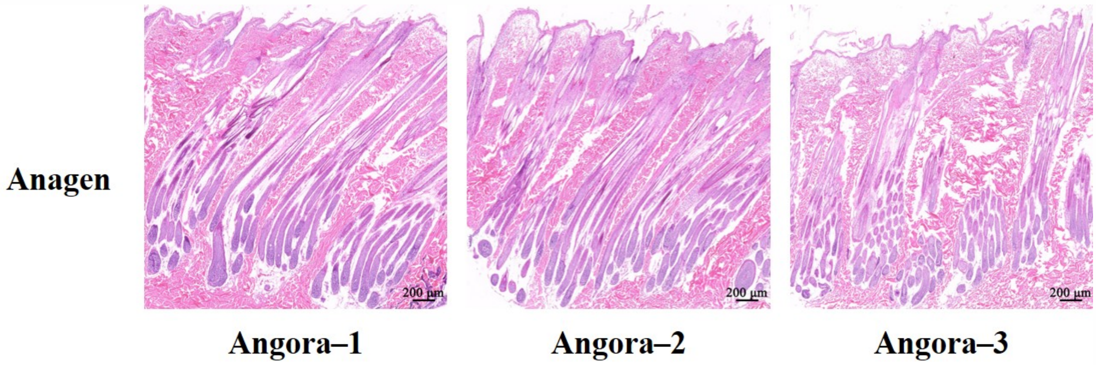

The HE staining results of the back skin of 3 independent Angora rabbits (Angora-1, Angora-2, Angora-3) showed that HF was in the Anagen state, the structure of HF was complete, and the hair bulb was deeply implanted in the dermis. Scale bar: 200 μm.
